# Traffic Stream Analysis by Radar Sensors on Two-Lane Roads for Free-Moving and Constrained Vehicles Identification

**DOI:** 10.3390/s23156922

**Published:** 2023-08-03

**Authors:** Giuseppe Cantisani, Giulia Del Serrone, Raffaele Mauro, Paolo Peluso, Andrea Pompigna

**Affiliations:** 1 Department of Civil, Constructional and Environmental Engineering, University of Rome La Sapienza, Via Eudossiana 18, 00184 Rome, Italy; 2 Department of Civil, Environmental and Mechanical Engineering, University of Trento, Via Mesiano 77, 38123 Trento, Italy

**Keywords:** traffic radar sensors, two-lane roads, free-moving and constrained vehicles, leader-follower influences, vehicle platoons, exponential headway model, apparently and actually conditioned vehicles

## Abstract

This paper focuses on the analysis of traffic streams on two-lane highways, which are crucial components of transportation networks. Traffic flow measurement technologies, such as detection stations, radar guns, or video cameras, have been used over the years to detect the level of traffic and the operating conditions. This type of sensor can record a large amount of data which is useful to evaluate and monitor road traffic conditions, and it is possible to identify free-moving and constrained vehicles by processing the collected data. This study introduces an exponential headway model to identify the headway threshold above which vehicles can be considered as unconditioned. However, this value could identify vehicles which still retain some autonomy in their speed and maneuvering. Therefore, an additional criterion to distinguish between apparently and actually conditioned vehicles has been introduced, analyzing the speed differences between a vehicle and the preceding one. Three-month sequences of traffic monitored through radar devices placed on some Italian two-lane roads have been analyzed and an exponential headway model has been introduced, as an illustrative example. The results show that introducing the criterion of maneuvering freedom can significantly improve traffic flow analysis, modifying the starting critical values of 4 and 8 s per each studied section, to 2.5 and 3 s, approaching the values suggested by international manuals for traffic flow quality analysis.

## 1. Introduction

Two-lane highways are a crucial part of the road network in many countries and are widely used for both systematic and non-systematic daily travel. Often, these roads make up the most extensive sub-network in a country’s transportation system, enabling higher-level infrastructures, such as freeways, railways, ports, and airports, to be reached even in the most remote areas [1]. As urbanization spreads and traffic demand increases, two-lane highways face new challenges that require innovative and cost-effective solutions to improve operational and safety performance [2]. This goal can be achieved through the access to high-resolution data encompassing those recorded by the widespread of cost-effective traffic monitoring systems, such as radar sensors [3]. The traffic control units are local traffic controllers which generate a complete overview of traffic, weather and road conditions, using the sensors connected to it [4]. Advanced signal processing algorithms, indeed, provide detailed and precise data, regardless of the sensing technology employed (traffic sensors: inductive, sunk into the road, or microwave, installed above the road; video cameras: numberplate recognition and Closed-Circuit TeleVision (CCTV); weather sensors, etc.) [5]. In addition to the advanced technological aspect, these sensors are easy to install on the roadside, non-intrusive, high performance, low power, and provide a large amount of information to be processed. These characteristics make them advantageous systems for monitoring the network. Thus, researchers can conduct comprehensive analyses of driver behavior in diverse traffic and road conditions over extended time periods.

One of the critical issues of two-lane highways is that passing occurs in the opposing lane of traffic, which has serious implications for traffic operation and safety. The limited passing opportunities result in a high level of interaction between vehicles, especially with the slow-moving ones. Therefore, it is essential to develop comprehensive models and metrics for performance analyses on this type of road, and to identify deficiencies and improve performances. Car-following models, within which each vehicle tries to maintain a desired time headway and speed behind the lead vehicle, attempt to simulate these interactions: drivers, in fact, continuously adjust their speeds based on their perceptions of the surroundings, the proximity to the desired speed, and the dynamics of the relationship with the lead vehicle [6]. The possibility of maintaining the desired speed depends on the presence of slower vehicles and on the option to overtake using the opposite lane. This interaction is particularly acute when both traffic flow and the percentage of slower vehicles increase, leading to vehicle platoons [7].

Understanding platooning behavior is crucial to modeling and analyzing traffic flow on two-lane highways accurately, as platoons can significantly affect operational performance and traffic flow. A platoon can be defined as a group of vehicles that travel together on the road, where following drivers adjust their speed, spacing, and acceleration according to the lead vehicle. As a result of these behavior dynamics, vehicles in the traffic stream can be grouped into two categories:

- A single vehicle or a platoon leader, which maintains a sufficiently large spatial and temporal headway with respect to the preceding vehicle, avoiding any influence from it and be able to travel at its desired or maximum speed at that moment.
- A platoon follower, which is unable to proceed at the desired speed and adjusts its driving behavior to follow the preceding vehicle (the platoon leader or another platoon component), while maintaining a reduced spatial and temporal detachment under its influence.

Passing lanes can mitigate the platoon impact on two-lane highways with high traffic levels, but they are not always available. For this reason, drivers may become frustrated and tend to accept smaller gaps in the opposing traffic to perform risky passing maneuvers, which can lead to head-on collisions. The average time that all vehicles delayed while traveling in platoons due to their inability to overtake was introduced in the 1985 edition of Highway Capacity Manual (HCM) [8] in terms of headways less than critical values. Even though threshold values have changed over the years and a new concept of Follower Density (FD) was introduced in the last edition [9], the threshold time headway for characterizing a vehicle in a non-free condition, i.e., constrained in a platoon, is a crucial element for analyzing two-lane roads according to the HCM.

**Figure 1:**
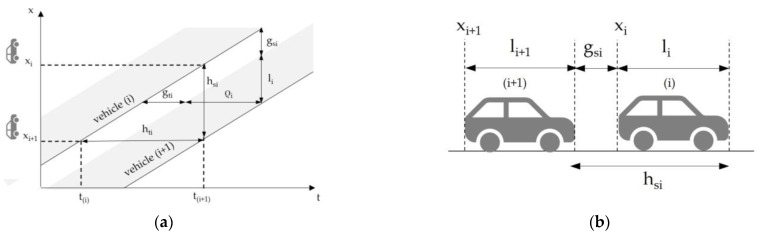
Time (**a**) and space (**b**) headways.

Time headway is a common parameter for studying vehicle behavior in a single-lane traffic stream, and the threshold value is an extremely widespread approach to distinguish free-moving vehicles from platooned vehicles. As shown in Figure 1, the time headway ht_i_ of vehicle i + 1 is defined as the absolute passing time of vehicle i + 1 minus the absolute passing time of vehicle i, traveling in front of vehicle i + 1 (a); ht_i_ is the sum of the time gap gt_i_ and the occupation time ρ_i_, where gt_i_ is the time between the rear of the lead vehicle and the front of the following vehicle and ρ_i_ is the time for the entire vehicle to pass the section. Headway can also be referred to in terms of distances between vehicles (b), and this is called spatial headway or spacing hs_i_, which is the distance from the front of one vehicle to the front of the previous one; hs_i_ is equal to space gap gs_i_ in addition to the length of the vehicle l_i_.

Different studies have proposed critical headway values to define vehicles that can travel at their desired speeds under free-flowing conditions. These vehicles are important for various applications in traditional traffic analyses and intelligent transportation systems [10,11]. Among all the works reviewed extensively in the following section, we can identify that a wide range of thresholds fall between 2 and 9 s. In this variety of works and values, we can distinguish two approaches to identify the threshold time headway in a single-line traffic stream, which refer to two different objectives and can be swayed toward two opposite extremes:

- The ones oriented toward identifying the Level Of Service (LOS) through the calculation of a Percentage of Time Spent Following (PTSF) in a platoon, the FD of vehicles in platoons, or even the Percentage of Followers (PF) in platoons. A not quite high value has to be taken, to avoid penalizing the analysis of traffic conditions by assigning the role of follower vehicles to those that are not actually impacted in their movements.
- The ones oriented toward defining Free-Flow Speed (FFS) or Operating Speed (V_85_), setting a threshold to be used as a filter to screen conditioned vehicles and select only those that can be considered in free movement. In this case, the value must be not quite low, to avoid considering vehicles that are included within a platoon.

To date, the literature does not clearly distinguish between these two tendencies toward which the search for the threshold value for the analysis of platoon formation can deviate, according to the specific needs that guide the research. In this context, the purpose of this paper is to further investigate the role of the headway threshold and emphasize the significance of selecting an appropriate value, which may vary based on local circumstances (geographic location, territorial context, road geometry, general and specific regulations, driving habits, etc.) and the specific operational needs.

For these reasons, the paper introduces the use of low-cost traffic monitoring systems, specifically radar sensors, to gather high-resolution information on traffic behavior and provides insights into vehicle interactions on two-lane highways, enabling a deeper understanding of traffic flow characteristics and deficiencies. The paper introduces a two-fold approach to characterizing platoon formation using threshold headway values. By employing an exponential headway model, the study identifies headway thresholds that distinguish between constrained and non-constrained traffic situations useful for FFS or V85 analysis. The addition of an extra criterion, analyzing speed differences between vehicles, enhances the accuracy of identifying conditioned vehicles, and thus improves LOS analysis. The paper demonstrates the effectiveness of the proposed methodology by applying it to real-life traffic data collected from two study road sections in the Veneto Region of Italy. The practical implementation of the analysis process, including the identification of threshold values through statistical tests, validates the proposed approach’s applicability in examining vehicle sequences.

The structure of the paper is as follows: Section 2 presents a comprehensive literature review on both road sensor technology for monitoring vehicle traffic and vehicular conditioning, summarizing the findings from the last 60 years of research on the headway threshold for free-moving vehicles. Section 3 outlines the methods used to characterize free-moving and conditioned vehicles and introduces the concepts of actual and apparent conditioning. Section 4 describes the research materials, including the characterization of vehicle traffic data using radar sensors, and identifies the specific test sections located in the Veneto Region of Italy. Section 5 provides a concrete example of the application of free-moving and constrained vehicles analysis, incorporating the headway threshold and maneuvering freedom criteria. Finally, Section 6 summarizes the study’s conclusions.

## 2. Literature Review

### 2.1. Road Sensor Technology for Monitoring Vehicle Traffic

The management and monitoring activities of a road network require managers to collect data regarding the operational and actual conditions of the infrastructure. To date, scientific literature indicates that there are several data collection techniques, which can be briefly distinguished into static and dynamic ones [12]. The first technique uses devices installed in a fixed section of the road, and information, such as speed, volume, and traffic density, are collected for the entire vehicular flow [13,14]. In contrast, the second method consists mainly of probe vehicles, which have on-board GPS devices capable of sending real-time and high-frequency data about their motion [13]. It is shown that probe vehicles have the advantage of providing travel information along the entire road infrastructure; however, this information comes only from vehicles equipped with innovative devices, while the static technologies are limited to the survey section but provide information on the entire vehicular fleet [15,16].

### 2.2. Vehicular Conditioning

As stated in the Introduction section, a considerable amount of research has been conducted in the last 60 years to determine the threshold headway that identifies free-moving vehicles and separates constrained and non-constrained traffic situations for vehicles traveling on the same lane. Below, we present a chronological review of the main works that have addressed this issue, outlining the primary objectives of each study and the threshold values identified or suggested over time.

Miller (1961) [17] proposed a random queue model, using data from the Swedish State Roads Institute collected on a straight section of a two-lane road. The study concluded that free-flow conditions are established when the traffic flow rate is very low and vehicles can overtake freely, with a headway value of 8 s. When time intervals are less than 8 s, the study considered a second criterion based on the relative velocities between consecutive vehicles to determine constrained conditions in moving queues.

Wasielewski [18] examined a semi-Poisson time headway distribution model, suggesting a 6–8 s headway for two-lane roads and a 4 s headway for freeways.

Radwan and Kalevela (1985) [19] identified a headway greater than 6.5 s to classify non-following conditions between different classes of vehicles.

McLean (1989) [20] stated that drivers on two-lane roads are affected by the presence of the vehicle ahead of them for headways of 9 s or less.

Lam et al., (1990) [21] compared operating speeds on dry and wet pavements of two-lane rural highways in New York state. They filtered free-moving vehicles considering vehicles with a minimum time gap of about 6 s or those heading a platoon of vehicles.

In 1991, Pursula and Enberg [22] conducted an analysis of speed-flow relationships, time headways, and platooning of traffic on two-lane rural roads in Finland. They used the platoon criterion of a 5 s time headway between successive vehicles for characteristics and level-of-service estimation.

Bennet et al., in 1994 [23] investigated critical headways at 58 study sites in New Zealand using different techniques to find the threshold. They identified a value between 3.0 and 4.5, recommending a value of 4.5 s to ensure that few following vehicles are misclassified as free.

Gattis et al., in 1997 [24] studied the behaviors of motorists. They considered a threshold headway value of 5 s between vehicles for platoon formation. They reviewed other studies by Messer and Morrall (1983) [25], Hoban (1983) [26], Guell and Virkler (1988) [27] in which the headway time used to define delay at two-lane highways varies from 3.5 s to 6 s and observed that a headway greater than 3 s may indicate a reduced desire to pass.

Dijker et al., in 1998 [28] analyzed differences in car following between congested and non-congested flows on Dutch roads and identified a critical gap value of 3.5 s for passenger cars and 5 s for trucks.

Vogel in 2002 [29] introduced a method that allows for establishing a threshold for free vehicles and determining how strongly the speed of the lead vehicle influences the speed of the following vehicle at a given headway in urban areas. The results suggested a time headway of 6 s as optimal for distinguishing between free and following vehicles for roads where traffic operates under capacity.

Van As in 2003 [30] selected 3.5 s as the threshold value to separate followers from platoon leaders on two-lane undivided roads in South Africa. Fifty percent of following vehicles have a headway of less than 3.5 s, 80% less than 4 s, and 99% less than 6 s.

Susarak et al., in 2004 [31] introduced platoon characteristics to measure traffic circulation quality on Tomey Express near Tokyo. They investigated new platoon criteria and platoon behavior and determined a critical headway relative speed method and exponential model. Based on the results on 18 sites, they defined a headway threshold of 3 s for passenger cars and 4 s for heavy vehicles.

In 2005, Fitzpatrick et al. [32] calculated free-flow speed at 79 tangent sites in suburban/urban and rural areas in six U.S. states by considering free-flowing vehicles as having at least a 5 s headway.

In 2005, Tseng et al. [33] determined the free-flow speed on Taiwan’s rural and suburban highways by considering vehicles traveling with a headway greater than 5 s as vehicles in free-flow condition.

Figueroa and Tarko in 2005 [34] identified a headway greater than or equal to 5 s as the point at which vehicles travel in free-flow conditions on the tangent segment and horizontal curve, considering 158 spots on U.S. two-lane rural highway segments.

Al-Kaisy and Karjala in 2008 [35] presented an empirical investigation into performance indicators on two-lane rural highways using field data from four study sites in the state of Montana. They calculated the free-flow speed by averaging the speed of all free-moving vehicles traveling with headways greater than 8 s, and the percent of follower vehicles was calculated considering vehicles traveling with headways less than 3 s.

Al-Kaisy and Durbin in 2009 [36] investigated vehicular platoons on two-lane highways in Montana. The results showed that the increase in speeds is more notable at short headways, and it diminishes when headways reach a value in the range of 5 to 7 s.

Lay in 2009 [37] suggested that drivers are affected by the presence of the vehicle ahead for headways of 9 s or less. Thus, he argued that the critical headway is still a matter of some debate with values quoted between 2.5 and 9 s, and with a best estimate of 4 s, and most in the range from 3.0 to 4.5 s.

Al-Kaisy and Karjala in 2010 [10] examined car-following interaction and the definition of free-moving vehicles on two-lane rural highways at eight study sites in Montana State. Empirical observations suggest that the car-following interactions on two-lane rural highways generally cease beyond a headway value of 6 s.

Hashim in 2011 [38] analyzed speed characteristics on two-lane rural highways in Egypt and investigated the relationship between 85th percentile speed and headways. A headway value of 5 s revealed the threshold between constrained vehicles and free-moving vehicles.

Lobo et al., in 2011 [39] conducted a study on Portuguese two-lane rural roads to define a gap value between two successive vehicles from which the vehicles can be considered as traveling in a non-platoon condition. They found a free gap of 6 s and highlighted the possible differences that may occur from site to site as a reflection of road geometry and from area to area (city, state, or country) as a function of general driver behavior.

In 2013, Semeida [40] posited that vehicles travel in free-flow conditions with a time headway of at least 8 s when developing new models to evaluate the level of service and capacity of rural multi-lane highways in Egypt.

Robertson et al., in 2014 [41] collected and studied traffic from different sites on freeways and multilane highways within the state of Texas for Level of Service models. Analyzing data from radar devices, they considered no free-flow conditions if the headway is 5 s or greater.

In 2015, Penmetsa et al. [42] studied two-lane intercity highways under mixed traffic conditions in India. It was assumed that vehicles traveling in the same lane with a relative speed of 2 km/h or less were in the car-following model. The gap corresponding to a 50% probability of not following was chosen as the critical gap, which was found to be 2.6 s; a following probability of about 80% considers vehicles with a headway of less than 4 s and 100% less than 5.5 s.

Boora et al., in 2018 [43] identified a headway value of 10 s for free-flow conditions. They introduced a headway and relative speed distribution analysis to define a second criterion for following behavior based on the speed difference between consecutive vehicles. A range of −7 to +15 km/h for the speed difference between the leader and follower was defined by data to identify followers and non-followers among vehicles with a headway of less than 10 s. Then, considering an acceptance curve method and a probability of 50%, a critical headway of 4.2 s was indicated.

Saha et al., in 2019 [44] investigated platoon phenomena on two-lane highways with heterogeneous traffic in northeast India to establish a headway threshold for free-moving vehicles. Their investigation indicated a value of about 6 s under which vehicles form platoons and that few drivers move in a platoon by choice even if they have a gap of more than 6 s and passing opportunities.

Al-Kaisy et al., in 2019 [45] presented an empirical investigation into car-following interactions from 15 study sites in Idaho, Montana, and Oregon. They argued that more aggressive drivers may start to interact with the lead vehicle and adjust their speeds when they are very close to the lead vehicle, while on the other hand, more cautious drivers may start to interact with the lead vehicle and adjust their speeds at a relatively large distance from the lead vehicle. Values for the critical headway are expected in a range that includes aggressive and cautious drivers, are site-specific, with a lower limit generally between 1 and 2 s and an upper limit between 6 and 7 s. Calculating the number of following vehicles in the whole transition range, they identified the cut-off value for platooning as the value to which an equivalent number of vehicles with smaller headways corresponds. Cut-off values from 15 sites range between 1.98 s and 3.85 s, and the author finally indicated a comprehensive critical headway value for identifying a vehicle in a following status as 2.5 s. The research work by Al-Kaisy et al. [45] was included in the project NCHRP 17-65-Improved Analysis of Two-Lane Highway Capacity and Operational Performance [25], a project approved by the TRB Committee on Highway Capacity and Quality of Service (ACP40) in 2019 for inclusion in the HCM.

**Figure 2:**
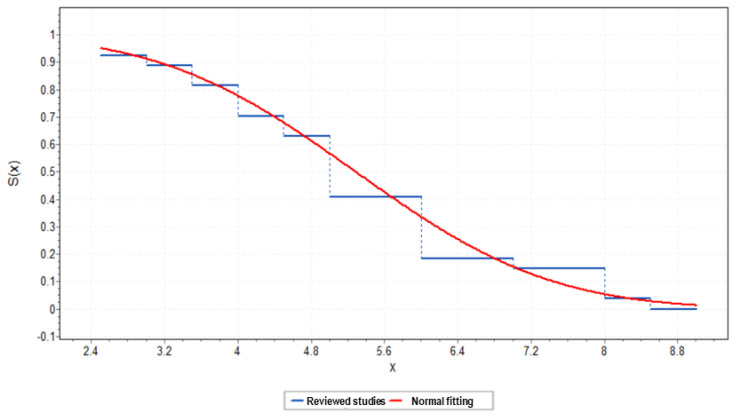
Survival function of the critical headway values for the platoon formation threshold identified in the reviewed studies: sampled and normal fitted trends.

Figure 2 depicts a graphical representation of the critical headway values for the platoon formation threshold identified in the reviewed past studies, ranging from a minimum of 2.5 s to a maximum of 9 s. The graph shows the survival function trend based on the literature values, which is the probability trend of finding values greater than or equal to a certain headway in the examined literature works. The continuous function is the trend of the survival function of the normal distribution that fits the data, with a mean of 5.29 s and a standard deviation of 1.68 s.

At this point, it is crucial to consider the various editions of the HCM and their distinct characteristics concerning the formation and impact of platoons on traffic quality and Level of Service determination for two-lane roads. The 1985 edition of the HCM [8] introduced the concept of Percent Time Delay (PTD), which stands for the percentage of time all vehicles are delayed while traveling in platoons due to their inability to overtake. However, obtaining real-time PTD measurements is impractical; therefore, the percentage of vehicles that travel behind a platoon leader at speeds lower than their desired speed and at headways less than a critical value is often used as a surrogate measure to determine the percentage of delays. The initial threshold introduced by the HCM was 5 s, which was maintained in subsequent editions of 1994 [46] and 1997 [47] guidelines for two-lane roads. In the 2000 edition of the HCM [48], PTD was renamed PTSF, which represents the freedom to maneuver, comfort, and convenience of travel by quantifying the percentage of travel time that vehicles must travel in platoons behind slower vehicles due to the inability to pass. The surrogate measure for PTSF is the percentage of vehicles traveling with headways less than a threshold. In this case, the suggested critical value was lowered to 3 s. This critical threshold value of 3 s remained unchanged in subsequent editions of 2010 [49] and 2016 [50], until the latest edition of 2022 [9], which presents a new methodological framework to analyze the quality of service of two-lane highways based on the Al-Kaisy et al. [45] and NCHRP 17-65 [25] studies. The HCM 2022 methodology [9] introduced FD as a new service measure, which is described as the number of vehicles in a follower state per mile per lane. FD overcomes the previous issues with PTSF performance measure since it can be directly measured in situ. In this context, a follower vehicle is defined by a headway equal to or less than 2.5 s, and FD is the service measure for all two-lane highway configurations.

## 3. Methods: Characterization of Free-Moving and Conditioned Vehicles

### 3.1. Main Aspect of Vehicular Conditioning and Exponential Headway Model

As mentioned earlier, single-lane traffic flow vehicles can be classified as either:

- Free-moving, unconstrained, or unconditioned vehicles, which are not in a platoon or are at the head of it and can travel at their desired speed with enough space to maneuver within the traffic flow;
- Constrained or conditioned vehicles within a platoon that must adjust their speed to that of the vehicle in front of them, potentially traveling at a slower speed than the desired one and are unable to overtake.

The headway, which is the time interval between two vehicles, is a common parameter used to characterize the two types of vehicles on a two-lane road. An often-used approach is to identify a critical time interval as the threshold for separating a conditioning situation from a non-conditioning one. There are two main types of models that have been used to represent single-lane time headway distributions: Simple models consisting of a single statistical distribution of observed headways, and models that are a set of two distributions, representing headways of constrained and free-moving vehicles in appropriate proportions [11,51].

The main simple models for temporal headway representation include Negative Exponential, Translated Exponential, Erlang, and Lognormal distributions [11,51,52]. Among these, the Negative Exponential distribution is suitable for modeling low-density traffic streams distributed without platoons along the road axis. In this case, the maximum degree of randomness is associated with the arrival of vehicles. If we consider a vehicle arrival process in a monitoring section that meets the following conditions:

1. Absence of memory: the number of arrivals in any time interval is independent of that in any other time interval.
2. Ordinariness: the probability of two or more arrivals occurring in a small-time interval is negligible compared to the probability of a single arrival occurring.
3. Stationarity: the arrival intensity λ is constant over time.

the probability that m arrivals will occur in a time interval Δt follows a Poisson vehicle arrival process, see Equation (1):

(1) $$P_{m}=\frac{a^{m}}{m!}e^{-a}$$

where:

m = 0, 1, 2, …, n, …

a = λ Δt(2)

If Equation (1) is fulfilled, the inter-arrival times between arrivals are exponentially distributed with a probability density function given by Equation (3):

(3) $$f(\tau )=\lambda e^{-\lambda \tau }$$

However, the Negative Exponential distribution provides non-zero probability values even for very small, almost zero, headways, which contradicts the fact that vehicles have non-zero lengths. To address this inconsistency, it is possible to assume the translated exponential as the probabilistic model for headways, which includes the smallest possible headway value under the examined traffic conditions [11,51,53].

In the presence of even small headways characterized by low frequencies, as well as an increase in mutual interferences between vehicles, an Erlang distribution can be assumed, which is a special case of the Pearson type III distribution [54]. The Erlang distribution is the probabilistic model for the inter-temporal distance between the instants of two non-contiguous events, generally the (n)th and (n + k)th, of a Palm Event Flow (Limited Memory Flow, a generalization of a Stationary Poisson Process) [55,56,57,58]. However, the Erlang distribution has always been used in traffic engineering for temporal vehicle spacings, which are intervals between successive contiguous and non-contiguous passes. With appropriate choices of the k-parameter values, it is often possible to achieve good fits of the Erlang distribution to experimental traffic data obtained by observing various discharge regimes [11,54,58,59].

In a dense traffic stream, in which most of the inter-vehicular time between vehicles falls within a limited number of amplitude classes, a log-normal probability density function can realistically be assumed to interpret the headway between vehicles [11,60].

While simple models consider the full experimental range of headways, they do not provide as broad a view of the factors influencing the pattern of arrivals. However, they usually require from the data fewer parameters for estimations. Mixed models, such as Buckley’s Semi-Poisson Model [61], Cowan’s M3 and M4 models [52], and Branston’s Generalized Queuing Model [62], result from a random arrival pattern modification, on the assumption that the two physical factors that prevent traffic in a single lane from behaving as a random phenomenon are the constraints imposed by stability and safety considerations and the constraints imposed by the lack of overtaking opportunities.

The distribution resulting from the modification takes the general form for mixed models, as in Equation (4):

f(τ) = ϑ g(τ) + (1 − ϑ) h(τ)(4)

where f(τ) is the probability density function of all headways, g(τ) is the probability density function of following headways, h(τ) is the probability density function of free-moving headways, ϑ is the proportion of following vehicles, and (1 − ϑ) is the proportion of free-moving vehicles. Other alternative methods include a data-driven approach that achieves greater adaptability to experimental data by interpreting statistically emerging behaviors from real data without the need for a priori assumptions or probability laws for speeds and time headways, such as the Two-Lane Road Statistical Platooning Model (TLR-SPM) [2].

### 3.2. Actual and Apparent Conditioning

The exponential headway model assumes that the headways of free-flowing traffic or headways between each platoon group tend to distribute randomly and can be modeled as a negative exponential headway distribution. The three key points of this model are:

- If vehicle arrivals in the selected section are totally random, and the time spacing between them is distributed according to an exponential random variable, the counting process is Poissonian.
- The exponential model for headways, like the Poisson model for counts, conforms to traffic counts when traffic flow is low.
- If the traffic flow cannot be considered low, the arrivals are no longer random, and the headways no longer follow an exponential distribution.

According to the exponential model, free-moving vehicles have a distribution of inter-arrival times that seems to be consistent with the distribution of random arrivals. Thus, the lowest value for which the headway distribution is no longer approximated by a translated exponential represents the minimum headway for which vehicles no longer exhibit the characteristics of unconditioned vehicles. Since the hypothesis of arrival randomness cannot be rejected for inter-arrival time values equal to or greater than this minimum headway, the absence of conditioning with respect to the preceding vehicle cannot be rejected either.

If the above conditions are not met, i.e., for inter-arrival time values less than the minimum headway, according to the exponential model, we can say that arrivals are characterized by a lack of complete randomness, and thus we cannot consider them as free-moving. We can then say that, according to the exponential model, these vehicles appear as conditioned. If conditioning is expressed as a lack of randomness, it indicates the existence of some regularity in the arrivals at the examined section, which results in some dependence or uniformity of motion characteristics between a given vehicle and the one in front of it.

However, it should be noted that the exponential model is based on an aggregate analysis of headways and their distributions. It is interesting to know what occurs when the individual vehicle passage has a headway below the threshold identified in the aggregate analysis. According to the exponential model, it is no longer a free-moving vehicle, and subsequently could be designated as conditioned. This leads to another query: can we classify it as actually conditioned merely since it has a headway below the threshold and assume that this situation explicitly expresses the existence of an authentic effect on the vehicle’s movement by the preceding vehicle? This cannot be said with certainty. The vehicle, in fact, may arrive at the monitoring section with a headway below the threshold, but it still retains some maneuvering freedom over the preceding vehicle, and thus it can manage its speed autonomously without actual conditioning. Therefore, it is appropriate to refer to this phenomenon as apparent conditioning since it cannot be identified considering only the headways. Therefore, an additional criterion to observe no evidence of maneuvering freedom is necessary to assert that the vehicle appears to be actually conditioned. To sum up, a conditioned vehicle has a headway below a certain threshold, which consequently does not conform to the hypothesis of completely random arrival according to the exponential model; therefore, there are two different situations:

- An apparent conditioning situation, where the vehicle still has some maneuvering freedom compared to the preceding vehicle.
- An actual conditioning situation, where the vehicle exhibits a lack of maneuvering freedom compared to the preceding one. In the latter case, the vehicle tends to conform to the kinematic conditions of the preceding vehicle, resulting in the creation of a platoon.

### 3.3. Conditioning by Necessity and by Choice

The primary circumstances leading to actual conditioning and the formation of platoons include:

- The presence of traffic congestion.
- The presence of a slower leading vehicle.
- High density of opposing traffic, preventing overtaking.
- Specific prohibitions on overtaking.

In addition to these situations, which can be identified as actual conditioning by necessity, there are also instances in which a driver may spontaneously choose to assume a position of uniformity with respect to the vehicle in front of them. In these scenarios, the driver may maintain a specific following distance and speed that matches the vehicle in front of them. This can result in a platoon of vehicles with a more uniform speed and inter-vehicle spacing, known as following by choice or intentional platooning [10,43]. It is based on the driver’s autonomous decision and not on external factors, such as traffic congestion/interactions or overtaking impracticality/prohibition. Situations where drivers choose to closely follow another vehicle can occur when:

- Their desired speed is very close to that of the leader of the platoon.
- They conform to a specific driving style dictated by the road geometry or environmental conditions.

In general, a distinction between following by choice and following by necessity cannot be made by only analyzing the data of headways and maneuvering freedom (for example, by examining the relationship between the kinematic characteristics of two consecutive vehicles) available from traffic monitoring. Therefore, a more comprehensive analysis of traffic conditions and driving behavior is necessary to fully understand the reasons behind the formation of platoons and the spacing between vehicles.

However, we can differentiate between choice and necessity in terms of spatial spacing (referred to as spacing for brevity). In following-by-necessity situations, there may be a greater inclination for vehicles to maintain smaller inter-vehicle spacing in the hope of overtaking a slower vehicle as soon as possible, resulting in a more tightly spaced platoon of vehicles. Conversely, in following-by-choice situations, vehicles may maintain a larger inter-vehicle spacing and form a more loosely spaced platoon. Although the vehicles in these platoons tend to conform to a common driving style, resulting in a certain regularity of arrival times (conditioning) and reduced freedom of maneuver (effective conditioning), they do not necessarily influence each other.

It is worth noting that a larger inter-vehicle spacing may also be observed between vehicles in apparent conditioning, where the headway is below the threshold of the exponential model, but there is still some freedom of maneuver. In these circumstances, due to driving styles or strategies, drivers may temporarily maintain a larger safety buffer between their vehicle and the preceding vehicle, resulting in a more loosely spaced platoon.

## 4. Materials: Characterization of Vehicle Traffic Data by Radar Sensors for the Analysis of Vehicle Conditioning

The objective of this study bases the proposed methodology on the analysis and processing of traffic data acquired through a static method; the following sections detail the adopted data sample.

### 4.1. Survey Method and Traffic Data

Vehicles’ identification in the free-moving and constrained conditions has been carried out by analyzing data collected through the Automatic Statistical Traffic Detection System of the Italian national road network manager, ANAS SpA [63]. The ANAS Road network currently has an automatic statistical traffic detection system with about 1200 counting sections. The sensors used for counting and classifying vehicles and measuring traffic flows are based on advanced microwave technology. They are designed to be installed directly over the center of the monitored lane. Their low power draw makes them highly versatile in use since they can be powered by a photovoltaic panel as well as directly by the mains (230 V/50 Hz). The sensors work in both directions of travel and can thus detect vehicles traveling against the flow: it can therefore continue working in the case of overtaking, lane changes, open worksites, etc. A single cable connects the sensor to the local controller. The main technical specifications of the adopted sensors are listed in Table 1:

The sensors in the counting stations send data to a centralized monitoring system called PANAMA (Piattaforma ANAs per il Monitoraggio e l’Analisi), which then provides verification, processing, and validation of the acquired data through a series of computerized control processes. In addition, the ability of the sensors to detect and capture the actual passing through the survey section is evaluated by ANAS personnel through simultaneous overlapping observation of the control unit readings and videos of the vehicles passing through [64]. Each control unit provides a sample of detailed traffic data, of which an extract of the reported information is shown in Table 2:

For each detected data, it is possible to know the following information: Time reference, which is the time instant of data acquisition (day and hour); Thousandths of a second, which is the time that should be added to the Time reference to determine the time of the actual passage of the vehicle; Lane, which is the lane on which the vehicle travels; Direction, which indicates the ascending or descending direction of travel; Speed, which is the value of the recorded speed (km/h); Time Gap, which is the time in a given section between the passage of the rear of a passing vehicle and the head of the following vehicle (s); Headway, which is instead the time in a given section between the passage of the head of a passing vehicle and the head of the following vehicle (s); and Vehicle Class, which is the Famas 9 + 1 classification code to identify different types of vehicles. The classification scheme adopted by Famas is: 1 Motorcycle, 2 Car, 3 Car + trailer, 4 Van, 5 Truck < 7.5 m, 6 Truck > 7.5 m, 7 Truck-trailer, 8 Articulated, 9 Bus, 10 Other.

The PANAMA database is constantly growing with real-time data collected from various continuous monitoring stations. In this paper, a portion of this database is used for illustrative purposes, limited to two monitored sections for 3 months, with a detailed number of observations in terms of individual vehicle transits as follows.

### 4.2. Identification and Characterization of Test Sections

The proposed survey was carried out on the highway’s road network of the Veneto region managed by ANAS. In detail, the national roads n.14 and n.309 and the related traffic control units, Control Unit 218 (CU 218) and Control Unit 1333 (CU 1333), respectively, have been considered. The extraction covers the months of February, May, and August of a normal operating year to account for the different conditions determined by seasonal traffic variation cycles. Table 3 shows the number of the recorded observations for each control unit in the examined time periods:

The control units will be explained in detail below in terms of their location and the infrastructural and spatial context in which they are installed.

#### 4.2.1. Control Unit 218 (CU 218)—National Road n.14 (SS14)

**Figure 3:**
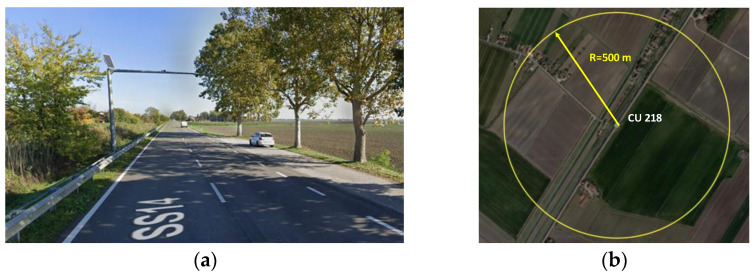
Control unit 218 on the national road n.14: (**a**) installation site with two lanes and lateral emergency stopping bay; (**b**) surrounding 500 m radius area (Google Earth).

The coordinates of the control unit 218, installed along the SS14, are given in the WGS84 reference system (lat. 45.59°, long. 12.47°). Figure 3a shows the installation site, which is a straight trunk, where it is assumed that the user behavior is not strongly influenced by the horizontal geometry of the infrastructure itself. At the monitoring point, there is a lateral emergency stopping bay situated adjacent to the travel lane. The satellite image in Figure 3b shows that the CU 218 is installed in a non-urbanized area with low residential density with predominantly agricultural activities. The surrounding 500 m radius area is characterized by few accesses to private areas and an intersection with a rural path, justified by the agricultural use of the local land.

#### 4.2.2. Control Unit 1333 (CU 1333)—National Road n.309 (SS309)

**Figure 4:**
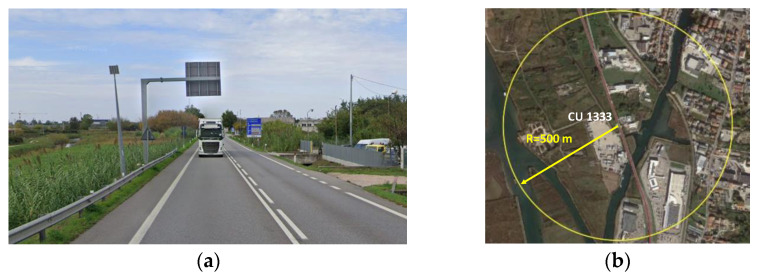
Control unit 1333 on the national road n.309: (**a**) installation site with two lanes; (**b**) surrounding 500 m radius area (Google Earth).

The coordinates of the control unit 1333, installed along the SS309, are given in the WGS84 reference system (lat. 45.19°, long. 12.27°). Figure 4a shows the installation site, which is even in this case a straight trunk, where it is assumed that the user behavior is not strongly influenced by the horizontal geometry of the infrastructure itself. The satellite image in Figure 4b shows that the CU 1333 is installed in an industrialized and agricultural area with medium residential density. The surrounding 500 m radius area is characterized by various accesses to different industrial activities, heralding a great influence of heavy vehicles on traffic operating conditions.

## 5. Results: Free-Moving and Constrained Vehicles Analysis

### 5.1. Headway Threshold Criterion for the Detection of Free-Moving Vehicles

In Section 3, we have observed the exponential model, which suggests that the arrivals of unconditioned vehicles can be described as Poissonian. Additionally, their inter-arrival time distribution follows a negative exponential distribution. When analyzing the experimental data described in Section 5.1, we can use the threshold headway criterion to identify free-flowing vehicles. This involves scrutinizing the inter-arrival times of the sampled vehicles in each monitoring section and identifying the lowest value for which the sample distribution can be approximated by an exponential distribution. This value is deemed as the threshold for the absence of conditioning: any inter-arrival values higher than the threshold are thus consistent with the distribution of random arrivals. Correspondingly, the associated sample headway distribution is considered as consistent with the distribution of random arrivals.

From a statistical standpoint, the threshold time value represents the minimum headway for which the hypothesis of absence of conditioning with respect to the preceding vehicle, or equivalently the randomness of arrival, cannot be rejected. In general, it can be observed that if f(τ) is an exponential probability density function and F(τ) is the related cumulative distribution function, the logarithmic transformation L(τ) of the complement to one of the F(τ) has a linear trend. Thus, to identify the critical headway, we can analyze the logarithmic transformation of the complement to one of the experimental headway distribution function $\tilde{F}\left(\tau \right)$, i.e., $\tilde{L}\left(\tau \right)=ln[1-\tilde{F}\left(\tau \right)]$, and we can examine the minimum time interval value τ*, for which $\tilde{L}\left(\tau \right)$ can be approximated by a straight line.

An explanatory example of how this can be carried out can be provided by using the traffic data collected according to the methods discussed in Section 5.1, which relate to the two monitoring sections presented in Section 5.2. Only values less than 300 s were considered for the two monitoring sections, under the assumption that arrivals with headways greater than this value were entirely random and not part of the same traffic stream. By grouping headway values less than 5 min into bins of 1 s (in order that the 0 s bin includes headways ≤ 0.5 s, the 1 s bin includes headways between 0.5 and 1.5 s, etc.), we can evaluate the experimental probability density function $\tilde{f}\left(\tau \right)$, the experimental probability distribution function $\tilde{F}\left(\tau \right)$, and the logarithmic transformation of its complement to one $\tilde{L}\left(\tau \right)$.

**Figure 5:**
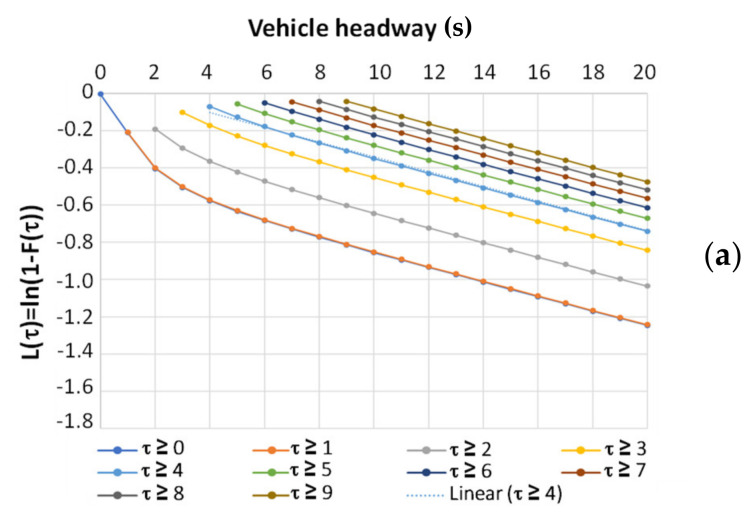

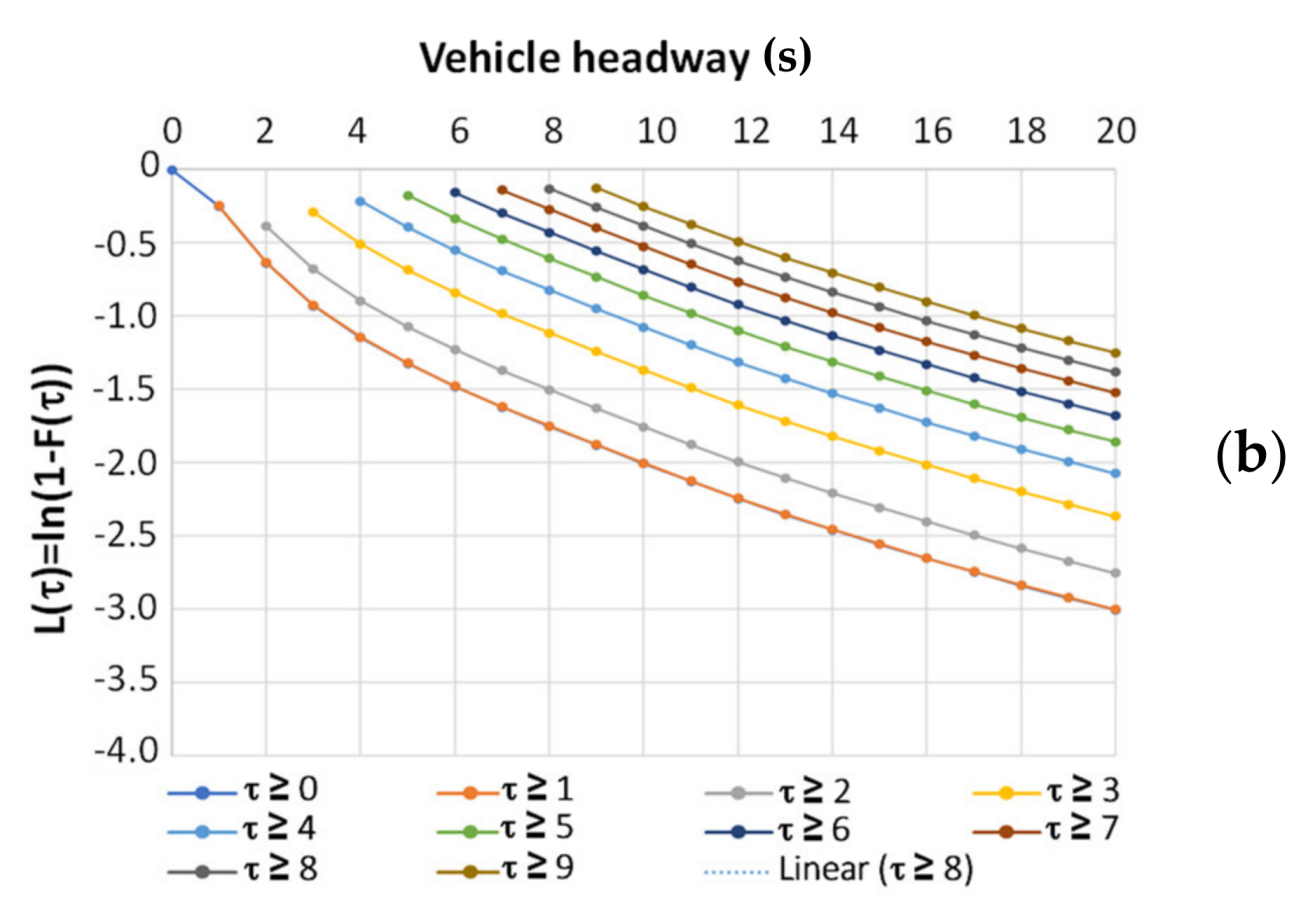
Experimental trend of $\tilde{L}\left(\tau \right)$ for $\tau \geq \hat{\tau *}$ with $\hat{\tau *}\leq \tau \leq 300$ s and $0\leq \hat{\tau *}\leq 9$ s: (**a**) control unit 218; (**b**) control unit 1333.

For each of the two considered monitoring sections, i.e., 218 and 1333, and directions of travel, after grouping the headways less than 300 s into bins of 1 s, we proceeded to test progressively every value of possible critical headway $\hat{\tau *}$ between 0 and 9 s. For every $\hat{\tau *}$, $\tilde{F}\left(\tau \right)$ was computed for $\hat{\tau *}$ ≤ τ ≤ 300 s. Thus, we analyzed the trend of $\tilde{L}\left(\tau \right)$ and estimated the regression line of the {X = τ; Y = $\left(\tau \right)$}. Upon analyzing the obtained trends, Figure 5 shows that as $\hat{\tau *}$ increases, the possibility of approximating $\tilde{L}\left(\tau \right)$ with a straight line significantly improves, with a Goodness of Fit (GoF) that can be initially observed merely graphically.

However, a graphical assessment of linearity as in Figure 5 does not allow us to identify the threshold value τ* as we intended. Measures of GoF, such as SSE (Sum of Squared Residuals) and R2 (Coefficient of Determination), were evaluated by considering the experimental trend of $\tilde{L}\left(\tau \right)$ for $\tau \geq \hat{\tau *}$ and that of the regression line. Additionally, the regression line obtained from each $\hat{\tau *}$ was employed to estimate the theoretical frequencies of each headway class for 1 s bins between 0 and 30 s. By considering the deviations between the sample frequencies and the theoretical frequencies obtained with the regression, MAPE (Mean Absolute Percentage Error) and MXAPE (Maximum Absolute Percentage Error) were also evaluated. The values for both sections, varying with τ*, are reported in Table 4:

Based on these clear trends, however, we are not yet able to identify which $\hat{\tau *}$ we can assume as the threshold value. It is necessary to develop a specific criterion that can be used to select the minimum value of $\hat{\tau *}$ that makes the linear approximation of $\tilde{L}\left(\tau \right)$ acceptable, and thus estimate the suitable $\tau *=\hat{\tau *}$ to represent the threshold value between free-flow and congested traffic. A criterion based on a statistical GoF test between probability distributions was introduced to address this question. The Kolmogorov–Smirnov (KS) test, commonly found in literature [65], was used to compare a sample distribution with a reference distribution (in this case, exponential). The KS test statistic D_n_, in Equation (5):

(5) $$D_{n}=max|\tilde{F}\left(\tau \right)-F(\tau )|$$

is obtained by comparing the experimental probability distribution functions, i.e., each sample distribution $\tilde{F}\left(\tau |\hat{\tau *}\;\leq \tau \leq 300\;s\right)$ with $0\leq \hat{\tau *}\leq 9$, and the theoretical probability distribution function with which the comparison is made, F(τ), at each point τ of the related sample. The null hypothesis H_0_ is that $\tilde{F}\left(\tau \right)=F(\tau )$. If the maximum difference between the theoretical and observed frequencies is large, we reject the null hypothesis for large values of $D_{n}$, while we do not reject it for small values of $D_{n}$. The critical value of $D_{n}$ (which we can denote as KSC) is defined for a certain sample size N and level of significance α, i.e., $KSC={KSC}_{\alpha ,N}$.

If $D_{n}\leq {KSC}_{\alpha ,N}$, then this result leads to the non-rejection of the null hypothesis H_0_, which states that the data come from the hypothesized distribution model, with the probability distribution function F(τ). Usually, a significance level of 5% (α = 0.05) is adopted as the reference value. In general, GoF tests, such as the KS test, provide some information about the fit between the sample and the theoretical model, based on *p*-values and critical values. In the case of the KS test, lower values of the statistic, particularly below the critical value, indicate an agreement between the sample and the theoretical model. While the meaning of the $D_{n}$ statistic is intuitively evident, calculating its probability distribution (under the null hypothesis H_0_) is more complicated. It is shown that under the null hypothesis H_0_, the probability distribution of the test statistic $D_{n}$ does not depend on the functional form of F(τ), and therefore the critical values can be obtained from specific statistical tables for small samples or calculated based on simple formulas for large samples, as a function of the significance level α. In general, the formula for ${KSC}_{\alpha ,N}$ is:

(6) $${KSC}_{\alpha ,N}=\frac{\sqrt{-0.5ln(\frac{\alpha }{2}})}{\sqrt{N}}$$

and if we consider the typical value of α = 0.05, then ${KSC}_{\alpha ,N}=\frac{1.36}{\sqrt{N}}$. However, in general, this type of test is extremely sensitive to the sample size. As the sample size increases, the *p*-values decrease dramatically, even in cases where the good agreement of data with the candidate model is evident [66]. As for ${KSC}_{\alpha =0.05,N}$, we see that it decreases rapidly as N increases. Considering the available sample sizes (in the order of several hundred thousand for each section), the KS test would lead to rejecting the exponential distribution for all critical headway values, even if they are sufficiently large, due to its intrinsic limitation. Despite the clear linear trend of $\tilde{L}\left(\tau \right)$ for $\tau \geq \hat{\tau *}$ as $\hat{\tau *}$ increases, due to the sample size N and the reduction in critical values ${KSC}_{\alpha =0.05,N}$, the KS test (as well as other similar GoF tests) provides extremely low *p*-values (and $D_{n}$ values much larger than excessively low critical values). This would always make the null hypothesis H_0_ of agreement with any theoretical model unacceptable [67,68,69,70].

In formulating the criterion to find the appropriate value of $\hat{\tau *}$ that can be assumed as the threshold value τ*, the problem is to make correct inference even in the case of a large N, by always applying the KS test to test the null hypothesis of exponential distribution of vehicle interarrival times greater than $\hat{\tau *}$. In literature, there are implementations of KS tests on large samples, including headways, by randomly extracting a large number of small sub-samples from the original sample [67,71]. Thus, in defining the criterion for choosing the threshold for free-moving vehicles, we have adopted this approach, based on random resampling without replacement from the initial sample of size N, by extracting a large number m of small sub-samples of size n ≪ N.

As previously mentioned, the choice to work with a big number (m = 1000) of small random sub-samples (n = 300) compared to the hundreds of thousands of headways in the original samples solves the problem of GoF test oversensitivity, related to the loss of power of hypothesis tests for very large sample sizes, that leads to rejecting significant distributions. Therefore, for each of the 1000 sub-samples, the $D_{n}$ statistic was calculated, and to evaluate the acceptability of H_0_ (exponential distribution) for each test threshold value τ* based on $\hat{\tau *}$, a significance level of α = 0.05 was chosen resulting in a critical value of ${KSC}_{\alpha =0.05,n=300}=0.0748$. The proposed criterion for assessing the overall GoF involves calculating the $D_{n}$ statistic for each sub-sample and obtaining the average $\bar{D_{n}}$ of these values. This criterion serves as a heuristic guideline to address the oversensitivity of traditional KS tests when dealing with large datasets. By comparing the mean value $\bar{D_{n}}$ with a critical value, it provides a basis for accepting or rejecting the null hypothesis for the entire dataset. Regarding the aforementioned KS tests, Table 5 presents the mean value $\bar{D_{n}}$ of the $D_{n}$ statistic over 1000 random sub-samples of size 300 for two illustrative cases. It also indicates (**) that the null hypothesis is not rejected based on the heuristic criterion ${\bar{D_{n}}<KSC}_{\alpha =0.05,n=300}$. By considering the smallest value of $\hat{\tau *}$ between 0 and 9 with a ${\bar{D_{n}}<KSC}_{\alpha =0.05,n=300}$, the critical headway value τ* is identified. Thus, using the exponential model in the two road sections for test, a threshold headway τ* of 4 s is identified in 218 and 8 s in 1333. Therefore, we can say that vehicles with a headway greater than or equal to these values appear in the sample with characteristics that allow us to attribute their arrivals to a degree of randomness compatible with a Poisson process. As a result, we can consider these as free-moving vehicles. Conversely, vehicles with headways less than these threshold values are not characterized by randomness of arrivals, and thus may express a certain conditioning.

### 5.2. Speeds and Manoeuvring Freedom Criterion for the Detection of Actual/Apparent Conditioning

We have highlighted that this status can be classified as actual conditioning if vehicles show a reduction in maneuvering freedom with a tendency to conform to the kinematic conditions of the preceding vehicle. On the other hand, it can be classified as apparent conditioning if the vehicles still retain a certain degree of maneuvering freedom compared to the previous vehicle. As previously stated, we need to further characterize this conditioning by introducing some other criteria that allow us to distinguish between actual conditioning and apparent conditioning. In order to ensure the accuracy of the analysis, a criterion that considers only the degree of maneuvering freedom of each vehicle must be established. Based on the information available for each passage reported in Section 5.2, this criterion can be derived from the analysis of the speed of each vehicle and its relative speed with respect to the preceding vehicle.

**Figure 6:**
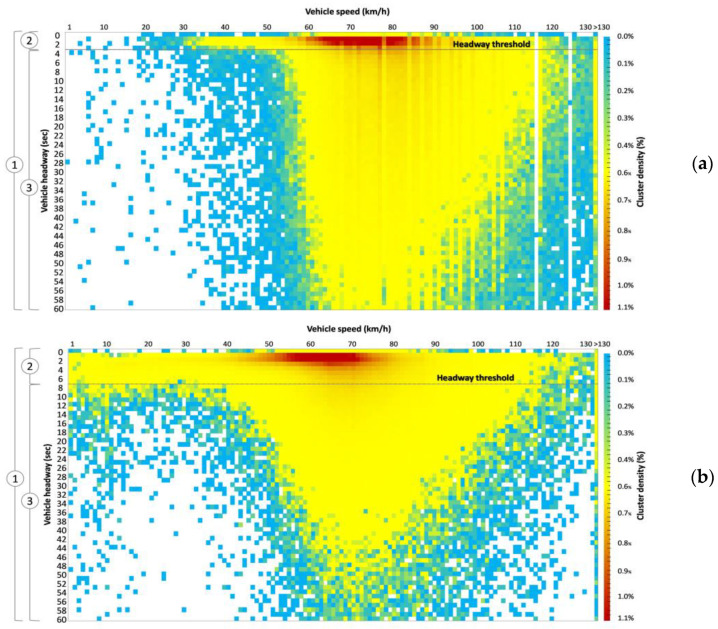
Experimental bivariate distribution of headway and speed-heat map for headways ≤ 60 s and speeds ≤ 130 km/h: (**a**) road section 218; (**b**) road section 1333. Three samples can be identified: (1) entire sample, (2) non-free vehicles, and (3) free-moving vehicles.

An initial analysis that helps to differentiate between free-moving vehicles and conditioned vehicles concerns the experimental bivariate distribution of headway and speed. This is shown in Figure 6 as a heat map for headways less than or equal to 60 s and speeds less than or equal to 130 km/h. From the experimental bivariate distributions of speed and headway, we can observe a separation between two distinct zones in the heat maps, which can be identified by the threshold between non-free (sub-sample 2) and free-moving vehicles (sub-sample 3). Below this threshold (i.e., 4 s for the CU 218 and 8 s for the CU 1333) the speed distribution covers a wider range that also includes values of 30–40 km/h, which are infrequent for headways above the same threshold.

**Figure 7:**
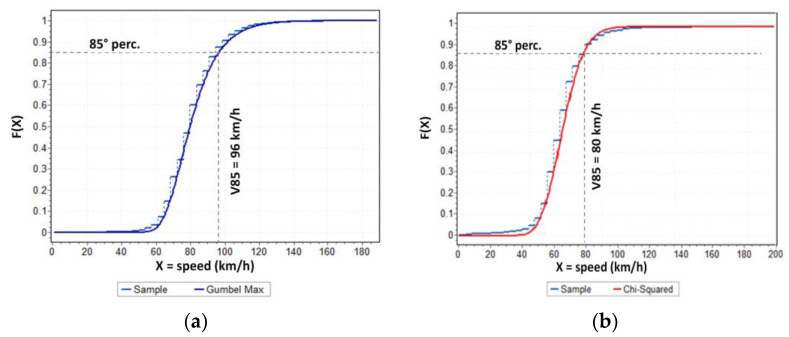
Sample and fitted cumulative distribution function for vehicle speeds within free-moving sub-samples: (**a**) road section 218; (**b**) road section 1333.

The threshold headway criterion, by itself, is insufficient in providing a comprehensive characterization of conditioning situations since it fails to distinguish between actual and apparent conditioning. Nevertheless, it facilitates the identification of unconditioned vehicles, and thus the threshold headway criterion establishes a value with a sufficient level of caution in filtering only fully and completely free-moving vehicles. Thus, free- moving vehicles in sub-sample 3 in Figure 6 can be filtered from the overall data using the headway threshold and further analyzed to determine speed percentiles in free-flow traffic conditions. For example, as introduced in the Introduction section, this can be useful in the analysis of operational speed (i.e., the 85th percentile of operating speed distributions or V_85_), that is a critical factor for road safety, since it significantly influences the frequency and severity of accidents [72,73], and it is widely acknowledged as a benchmark value for evaluating consistency in homogeneous road sections [74,75,76]. Figure 7 illustrates the experimental and best-fitting distributions (Gumbel Max for CU 218 and Chi-Squared for CU 1333) and the corresponding V_85_ values in the two monitored sections from the speed data in sub-sample 3.

**Figure 8:**
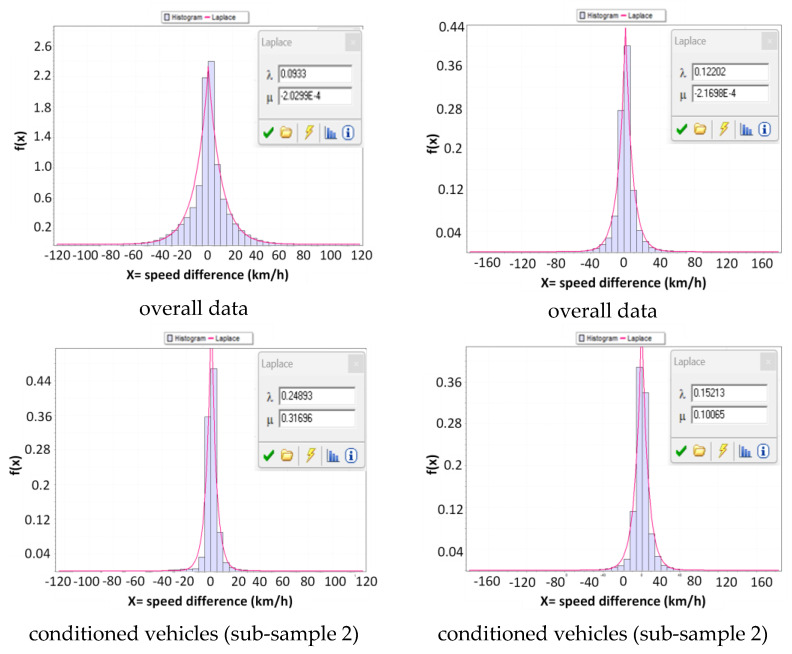

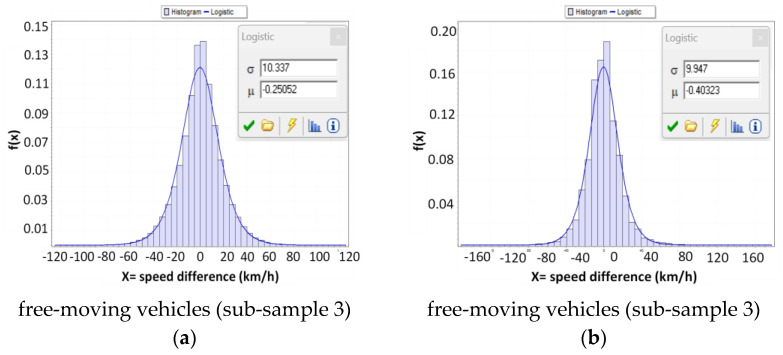
Experimental distributions of speed differences for the overall sample, sub-sample 2, and sub-sample 3: (**a**) road section 218; (**b**) road section 1333.

After distinguishing the two subgroups of free-moving and conditioned vehicles based on the threshold headway and identifying their differences in terms of speed distributions, further analyses can be conducted in relation to the relative speeds. The speed difference between a vehicle and the preceding one can be used as a parameter to represent the freedom of maneuver, where a lower absolute value implies a higher likelihood of the vehicle being influenced by the preceding one and vice versa. Figure 8 presents representative histograms of the experimental distributions of speed differences for the overall sample, as well as the two partitions of conditioned vehicles (sub-sample 2) and free-moving vehicles (sub-sample 3).

The figure also includes the distribution that best fits the experimental data based on the KS test. As evidenced by the images and numerical results in Table 6, the distributions appear substantially different in terms of both descriptive statistics and theoretical distributions that accurately describe their trends.

The descriptive statistics indicate that the mean values are close to zero in all cases, but the standard deviation values differ among the samples, with sub-sample 2 having values closer to the mean than the other two samples. The skewness values are close to zero, indicating that the distributions are approximately symmetrical. However, all kurtosis values are greater than 3, suggesting that all the distributions are more peaked than a normal distribution, with sub-sample 2 having a more peaked distribution than the other samples. These differences in kurtosis values suggest structural differences in the data distribution, primarily in comparison to sub-sample 3. These observations apply to both the CU 218 and CU 1333 test sections illustrated in Figure 8, which displays the histograms and the best-fit function.

The selection of the Logistic distribution for sub-sample 3 and the Laplace distribution for both sub-sample 2 and the overall data further indicates differences in the headway distribution between sub-samples 2 and 3, in terms of the presence of extreme values and the probability of producing values close to the mean. Therefore, speed differences in sub-sample 3 with headway values equal to or greater than the threshold value may be more prone to extreme variations than those in sub-sample 2.

Following Miller’s (1961) proposal [17], later adopted by Boora et al., (2018) [43], we can utilize these differences in distributions to identify the range of speed differences in which the frequencies of the non-free-moving vehicle sub-sample exceed those of the free-moving vehicle one. If the speed difference distribution of sub-sample 2 exceeds that of sub-sample 3 within a certain interval, then there are more vehicles in sub-sample 2 with speed differences falling within that interval than there are free-moving vehicles in sub-sample 3. This suggests that vehicles in sub-sample 2 with speed differences in that interval may have probabilistically different behaviors from free-moving vehicles in sub-sample 3. We can consider the vehicles in sub-sample 2 with speed differences falling within the interval that characterizes probabilistically different behaviors from free-moving ones as actually conditioned, and those with differences outside the interval as apparently conditioned.

**Figure 9:**
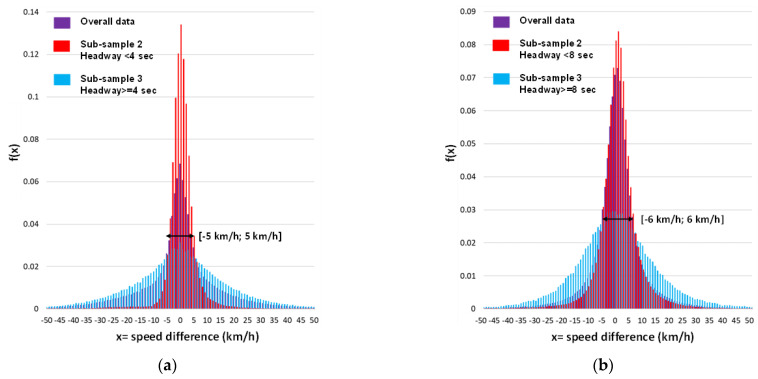
Experimental distributions of speed differences for the overall sample, sub-sample 2, sub-sample 3, and conditioning prevalence interval: (**a**) road section 218; (**b**) road section 1333.

Using this approach, as shown in Figure 9, we can identify the range of speed differences in which the frequencies of the distribution of conditioned situations (actual + apparent) surpass those of free-moving ones, representing a range of conditioning prevalence that can indicate actual conditioning. Based on this range, we can define a new criterion to represent the freedom of maneuver and to distinguish between these two situations:

- Vehicles with speed differences outside this range of conditioning prevalence are apparently conditioned;
- Vehicles with speed differences within this range of conditioning prevalence are actually conditioned.

In the case under consideration, the ranges that can be identified based on the experimental distributions, as shown in Figure 9, consider speed differences between −5 km/h and 5 km/h for road section 218 and between 6 km/h and 6 km/h for road section 1333.

### 5.3. Headway Acceptance Curves in Vehicle Conditioning

Therefore, we can consider that a vehicle is actually conditioned if it demonstrates:

- A headway value below the threshold identified with the exponential model (in the example, 4 s for CU 218 and 8 s for CU 1333).
- A speed difference with the preceding vehicle within the range of conditioning prevalence, identified based on the comparison between the experimental frequencies of speed differences among the conditioned sample (actual + apparent) and the unconditioned sub-sample (in the example, [−5 km/h, 5 km/h] for the road section 218 and [−6 km/h, 6 km/h] for the road section 1333).

**Figure 10:**
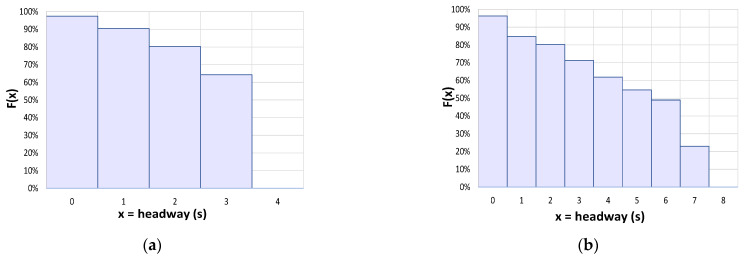
Histograms for the experimental density distribution of actual conditioning vehicles for headway class below the exponential model threshold: (**a**) road section 218; (**b**) road section 1333.

Thus, an apparently conditioned vehicle is a vehicle that, despite passing through the monitoring section with a headway lower than the threshold value, appears to have a reduced level of randomness, yet still exhibits a certain degree of maneuverability. This maneuverability is evident in the speed difference with the preceding vehicle, which lies outside the range of conditioning prevalence. By applying both criteria to the entire monitoring sequence, we can identify actual conditioning vehicles and analyze their percentage trend according to the headway class. In Figure 10, for CU 218, we can observe a decrease from 100% at 0 s headway to 90% at 1 s, 80% at 2 s, 65% at 3 s, and ultimately 0% at the threshold value of 4 s. Similarly, for CU 1333, we can see a decrease from 100% at 0 s to 80% at 2 s and progressively to 62% at 4 s, 48% at 6 s, 23% at 7 s, and ultimately 0% at the threshold value of 8 s.

**Figure 11:**
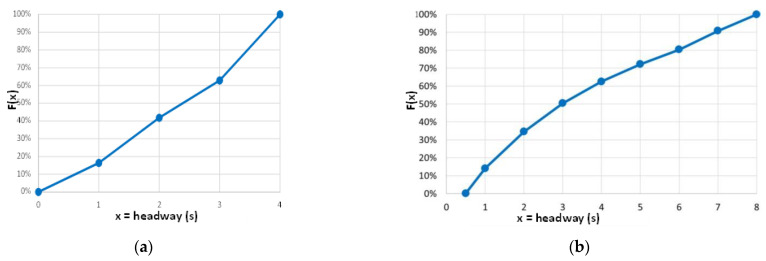
Experimental cumulative distribution of apparently conditioned vehicles with respect to the total number of conditioned vehicles (actual + apparent): (**a**) road section 218; (**b**) road section 1333.

As suggested by Boora et al. [43], we can plot the cumulative percentage of apparently conditioned vehicles, representing the cumulative probability of non-following vehicles. Figure 11 shows the results for the two test sections. For CU 218, a headway threshold value of 3 s includes approximately 60% of the apparently conditioned vehicles, while a threshold value of 2 s reduces this percentage to 40%. Similarly, for CU 1333, a threshold value of 6 s includes approximately 80% of the apparently conditioned vehicles, while values of 3 s and 2 s correspond to 50% and 35%, respectively. Configured as an acceptance curve [43], this plot can be used to identify a critical gap value, above or below which vehicles would travel as free or following vehicles, respectively. Considering the 50% probability value for each of the two cases, we obtain a critical value of slightly under 2.5 s for CU 218, and about 3 s for CU 1333. Despite differences in the threshold headway values of the exponential model, this range appears narrower and comparable to the threshold indicated by the latest editions of the HCM for determining measures of effectiveness (PTSF or FD) for analyzing traffic quality and Levels of Service.

### 5.4. Further Insights on the Distribution of Inter-Vehicle Distance in Vehicle Conditioning

An interesting aspect to investigate is certainly the spacing between vehicles, namely, the spatial distance between two consecutive vehicles. In Table 7, the trends of the average spacing (in meters) between consecutive vehicles for headway bins between 0 (τ≤0.5 s) and 10 (9.5≤τ<10.5 s) are shown. As we can see, the average spacing for the threshold headway is 87 m for CU 218 (τ* = 4 s) and 157 m for CU 1333 (τ* = 8 s). Since we consider vehicles with headways greater than t* as free moving, they correspond to average headways greater than 87 m for CU 218 and 157 m for CU 1333.

**Figure 12:**
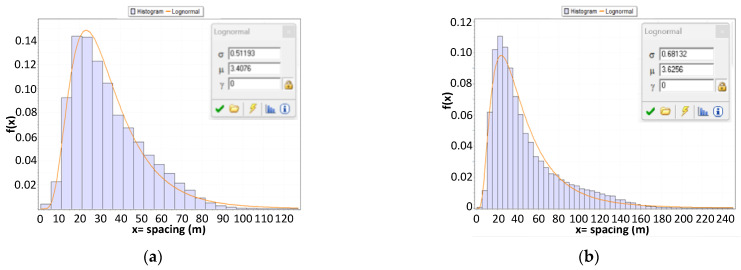
Histograms and lognormal fitting for spacing (m) in conditioned (actual + apparent) vehicles: (**a**) road section 218; (**b**) road section 1333.

If we shift our attention to analyzing the probability distribution of the spacing for vehicles below the threshold that we defined as conditioned simply due to the fact that they do not meet the criteria of the exponential model for free-moving vehicles (and thus composed of a combination of vehicles that are apparently and actually conditioned), we observe that the average spacing is 34.2 m, with a 90th percentile of 59 m for CU 218 (sample distribution and lognormal fitting in Figure 12a), and 47 m, with a 90th percentile of 99 m for CU 1333 (sample distribution and lognormal fitting in Figure 12b).

If we consider the two groups into which we can divide the conditioned vehicles, i.e., actually and apparently conditioned, based on the prevalence interval of the conditioning concerning speed differences, the analysis of distributions clearly highlights differences between the two clusters. In CU 218, the average spacing is 45.4 m for apparently conditioned vehicles, and it is reduced to 31.8 m for actually conditioned vehicles, with a standard deviation decreasing from 19.7 m to 15.4 m. In CU 1333, the average spacing is 64.4 m for apparently conditioned vehicles, and it is reduced to 41.2 m for actually conditioned vehicles, with a standard deviation decreasing from 40.2 m to 29.0 m. Consequently, we can confirm that the two groups exhibit statistically different behaviors, even in terms of spatial distancing.

**Figure 13:**
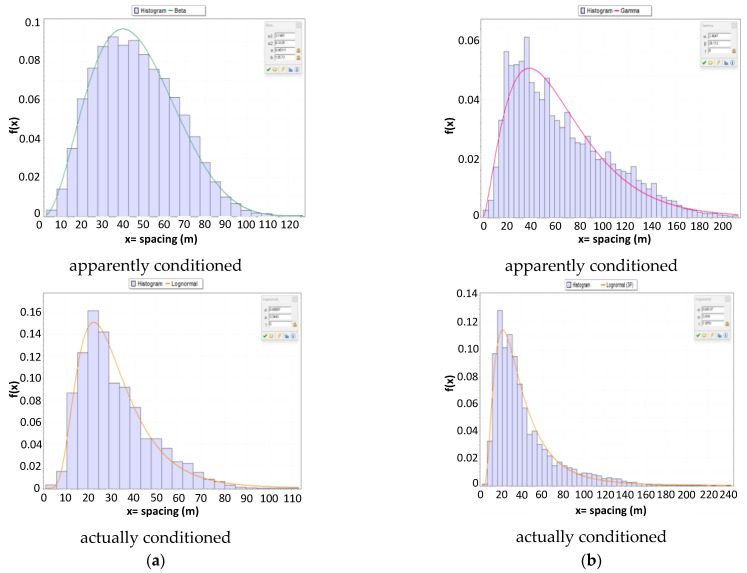
(**a**) Road section 218; (**b**) road section 1333.

Figure 13 presents the histograms for the two groups, i.e., apparently and actually conditioned, of spatial distances and the resulting distributions from the best approximation according to the usual KS test (lognormal for actually conditioned and beta for apparently conditioned in CU 218; 3-parameter lognormal for actually conditioned and gamma for apparently conditioned in CU 1333).

## 6. Conclusions

Two-lane highways are essential components of transportation systems in many countries, but they face operational and safety challenges due to limited passing opportunities and platooning behavior. The increasing availability of low-cost traffic monitoring systems, such as radar sensors, provides high-resolution information in various traffic and road geometry conditions, allowing for comprehensive analyses of vehicle behavior on this specific type of road. This enables a deeper analysis of vehicular interactions on two-lane highways, which is essential for identifying deficiencies and improving operational performance for this important category of roads.

The literature review highlighted the importance of threshold headway values, ranging from 2.5 to 9 s, in characterizing platoon formation and differentiating between constrained and non-constrained traffic situations. The exponential headway model helps to identify the headway threshold above which vehicles can be considered unconditioned due to the randomness of their arrivals, and statistical tests were used to determine the critical value. However, the threshold headway value, determined using the exponential model, could identify vehicles which still retain some autonomy in their speed and maneuvering. Therefore, the paper introduced as a novelty an additional criterion to distinguish between apparently and actually conditioned vehicles: the analysis of speed differences between a vehicle and the preceding one. Using this range as a criterion, a distinction was established between actual conditioning and apparent conditioning. This approach allowed for a more accurate assessment of traffic flow quality, without relying solely on the exponential model and avoiding the excessively cautious approach that may result from it.

The application of the proposed methodology to real-life sequences from two study road sections in the Veneto Region of Italy demonstrated the randomness of arrival thresholds in a wide range of values, which in the two specific cases were 4 s and 8 s, respectively and significantly differed from the thresholds of the most recent editions of the HCM, which is 2.5 s. Introducing the criterion of maneuvering freedom, critical values for platoon formation within a narrower range could be found. In fact, through the identification of headway acceptance curves for actual/apparent conditioning, critical headway turned out to be 2.5 s and 3 s, approaching the values suggested by international manuals for traffic flow quality analysis.

The findings of this study provided valuable insights into the characterization of vehicular conditioning on two-lane highways. Although the number of road sections studied in this article was limited, the findings served as an exploratory analysis of the proposed methodology. Future research could further investigate this methodology by collecting data using radar detection systems in a greater number of sections. These research opportunities could involve distinguishing local traffic and infrastructure situations to provide additional and in-depth contributions to the development of effective strategies for improving road safety and operational performance.

## Figures and Tables

**Table 1:** Technical specifications of the adopted sensors.

**Technology**	Microwave	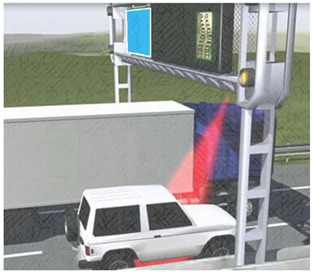
**Operating Conditions**	Temperature: −20 °C to +60 °C	
	Humidity: 0% to 100%, without condensation	
**Power**	12 VDC to 24 VDC	
**Consumption**	1 W approx.	
**Output Data**	Number, direction, speed, length, occupation time, headway and gap between vehicles, vehicle category.	
**Data Generated**	Time stamp in milliseconds	
	Sensor diagnostics	
**Dimensions And Weight**	165 × 95 × 280 mm (H × W × D)	
	1.8 kg without accessories	
**Enclosure Material**	ABS	
	Stainless-steel roof	
**Protection Rating**	IP66	
**Mounting System**	Collar/locking assembly	
**Mounting**	Height: 6 m to 7.5 m	
	Position: aligned with center of lane	

**Table 2:** Example of control unit traffic survey.

Time Reference	Thousandths of a Second	Lane	Direction	Speed (km/h)	Time Gap (s)	Headway (s)	Vehicle Class
01/02/2019 00:00:40	200	2	A	72	97.1	97.3	2
01/02/2019 00:01:12	300	2	A	76	31.9	32.1	2
01/02/2019 00:01:35	0	2	A	79	22.6	22.7	4
01/02/2019 00:01:36	900	2	A	90	1.8	1.9	2
01/02/2019 00:02:01	100	1	D	69	172.1	172.3	2
01/02/2019 00:03:32	300	1	D	92	91.1	91.3	7
01/02/2019 00:04:14	100	2	A	69	157.2	157.4	2
01/02/2019 00:04:33	100	2	A	86	18.8	18.9	2

**Table 3:** Number of recorded observations by the control units.

Months	CU 218	CU 1333
February	226,613	265,321
May	269,708	300,520
August	271,787	311,512
Total	768,108	877,353

**Table 4:** GoF measures for linear regression of the {X = τ; Y = $\left(\tau \right)$} values for sections 218 and 1333.

$\hat{\tau *}$	Section 218					Section 1333				
	$N(\tau \geq \hat{\tau^{*}})$	R2	SSE	MAPE	MXAPE	$N(\tau \geq \hat{\tau^{*}})$	R2	SSE	MAPE	MXAPE
0	363,375	0.9578	0.2026	5.22%	28.80%	441,177	0.9486	1.5639	17.54%	49.83%
1	362,114	0.9806	0.0733	3.11%	19.91%	440,176	0.9579	1.0497	14.39%	47.49%
2	294,183	0.9943	0.0178	1.70%	9.76%	343,460	0.9705	0.5978	11.59%	36.30%
3	242,699	0.9978	0.0060	1.06%	5.47%	233,076	0.9775	0.3781	9.73%	27.78%
4	219,188	0.9990	0.0024	0.71%	3.37%	174,223	0.9812	0.2650	8.50%	22.48%
5	204,321	0.9995	0.0011	0.51%	2.21%	140,319	0.9836	0.1963	7.58%	19.05%
6	193,053	0.9997	0.0006	0.38%	1.45%	117,540	0.9852	0.1493	6.78%	16.58%
7	183,559	0.9998	0.0003	0.30%	1.06%	100,536	0.9865	0.1148	6.06%	14.84%
8	175,411	0.9999	0.0002	0.24%	0.80%	87,319	0.9877	0.0877	5.39%	13.45%
9	167,998	0.9999	0.0001	0.21%	0.55%	76,490	0.9890	0.0657	4.76%	12.25%

**Table 5:** KS test for section with H_0_ considering $\tilde{F}\left(\tau |\hat{\tau *}\;\leq \tau \leq 300\;s\right)\;$ with $0\leq \hat{\tau *}\leq 9$ compatible with an exponential distribution for sections 218 and 1333.

$\hat{\tau *}$	Section 218		Section 1333	
	$\bar{D_{n}}$	$\bar{D_{n}}<{KSC}_{\alpha =0.05,n=300}\;(--=no;**=yes)$	$\bar{D_{n}}$	$\bar{D_{n}}<{KSC}_{\alpha =0.05,n=300}\;(--=no;**=yes)$
0	0.2599	--	0.1667	--
1	0.2187	--	0.2407	--
2	0.1687	--	0.2091	--
3	0.0956	--	0.1400	--
4	0.0443	**	0.1212	--
5	0.0711	**	0.1293	--
6	0.0655	**	0.1325	--
7	0.0698	**	0.0933	--
8	0.0636	**	0.0777	**
9	0.0738	**	0.0642	**

**Table 6:** Descriptive statistics and theoretical distributions for speed differences.

Section	Section 218			Section 1333		
Group	Overall Data	Sub-Sample 2	Sub-Sample 3	Overall Data	Sub-Sample 2	Sub-Sample 3
Mean	−2.0299 × 10^−4^	0.31696	−0.25052	−2.17 × 10^−4^	0.10065	−0.40323
Std. Deviation	15.157	5.6813	18.7S	11.59	9.296	18.042
Skewness	−0.06348	0.54154	−0.00456	0.1003	−0.32462	0.35945
Kurtosis	3.649	18.288	1.5158	13.244	19.841	4.4177
Fitting Function (best)	Laplace	Laplace	Logistic	Laplace	Laplace	Logistic

**Table 7:** Average spacing (m) for headway bins (1 s) for sections 218 and 1333.

Headway Bin	Average Spacing (m)	
(s)	Section 218	Section 1333
0 (0–0.5)	7	8
1 (0.5–1.5)	21	19
2 (1.5–2.5)	39	33
3 (2.5–3.5)	63	51
4 (3.5–4.5)	87	71
5 (4.5–5.5)	111	92
6 (5.5–6.5)	135	114
7 (6.5–7.5)	159	136
8 (7.5–8.5)	183	157
9 (8.5–9.5)	206	178
10 (9.5–10.5)	230	199

## Data Availability

The data presented in this study are available on request from the corresponding author. The data are not publicly available due to confidentiality issues and respect for privacy.
